# GEN1 from a Thermophilic Fungus Is Functionally Closely Similar to Non-Eukaryotic Junction-Resolving Enzymes

**DOI:** 10.1016/j.jmb.2014.10.008

**Published:** 2014-12-12

**Authors:** Alasdair D.J. Freeman, Yijin Liu, Anne-Cécile Déclais, Anton Gartner, David M.J. Lilley

**Affiliations:** Cancer Research UK Nucleic Acid Structure Research Group, MSI/WTB Complex, The University of Dundee, Dow Street, Dundee DD1 5EH, UK

**Keywords:** GFP, green fluorescent protein, TEV, tobacco etch virus, BSA, bovine serum albumin, BSA, bovine serum albumin, EDTA, ethylenediaminetetraacetic acid, DNA recombination and repair, Holliday junction resolution, *Chaetomium thermophilum*, FEN1, thermophilic proteins

## Abstract

Processing of Holliday junctions is essential in recombination. We have identified the gene for the junction-resolving enzyme GEN1 from the thermophilic fungus *Chaetomium thermophilum* and expressed the N-terminal 487-amino-acid section. The protein is a nuclease that is highly selective for four-way DNA junctions, cleaving 1 nt 3′ to the point of strand exchange on two strands symmetrically disposed about a diagonal axis. CtGEN1 binds to DNA junctions as a discrete homodimer with nanomolar affinity. Analysis of the kinetics of cruciform cleavage shows that cleavage of the second strand occurs an order of magnitude faster than the first cleavage so as to generate a productive resolution event. All these properties are closely similar to those described for bacterial, phage and mitochondrial junction-resolving enzymes. CtGEN1 is also similar in properties to the human enzyme but lacks the problems with aggregation that currently prevent detailed analysis of the latter protein. CtGEN1 is thus an excellent enzyme with which to engage in biophysical and structural analysis of eukaryotic GEN1.

## Highlights

• We have identified the gene for the junction-resolving enzyme GEN1 in the thermophilic fungus *C. thermophilum*.
• CtGEN1 is a nuclease that is highly selective for the structure of four-way DNA junctions.
• CtGEN1 binds to DNA junctions as a discrete homodimer with nanomolar affinity.
• Second-strand cleavage by CtGEN1 is accelerated to ensure productive resolution.
• In contrast to the human enzyme, CtGEN1 is highly suitable for structural and mechanistic investigation.

## Graphical abstract

**Figure:**
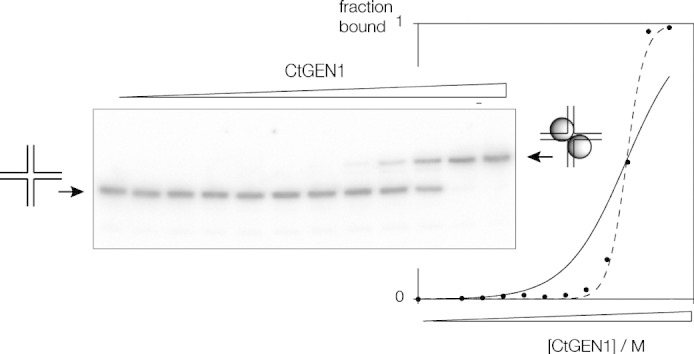

## Introduction

Homologous genetic recombination performs indispensable functions. In mitotic cells, it provides a mechanism for high-fidelity double-strand break repair and contributes to the resolution of interstrand crosslinks and lesions arising during replication. In meiosis, it enhances genetic diversity and ensures accurate chromosomal segregation by generating a temporary physical linkage between homologous chromosomes. Deficiency in homologous recombination leads to markedly elevated susceptibility to a variety of cancers.

The central intermediate in homologous recombination is a four-way DNA, or Holliday, junction [1], where four DNA helices are connected by the covalent continuity of the strands. The key event in the pathway is the processing of the junction. Holliday junctions formed in cellular chromosomal DNA are processed by two types of process, distinguished by whether or not they involve nucleolytic cleavage. The first process, called “dissolution”, requires the BLM helicase to translocate two adjacent junctions toward each other before they are unlinked by topoisomerase IIIα [2], [3], [4]. This pathway, which does not lead to strand exchange, is probably the primary response to the presence of DNA junctions in mitotically dividing cells, and defects lead to Bloom syndrome [5] that is characterized by genomic instability [6]. Any junctions that persist are processed by mechanisms that are based on the action of nucleases that are selective for the structure of a four-way DNA junction; this second process is called “resolution”. Junction-resolving enzymes have been well characterized in bacteria, phage, archaea and yeast mitochondria (reviewed in Ref. [7]).

In eukaryotic cells, there are a number of nucleases that can act upon branched DNA structures, but in the last 3 years, it has become clear that there are two main nucleolytic activities that are responsible for recognizing and processing of four-way DNA junctions. The first to be identified is GEN1 (Yen1 in yeast) [8], [9], [10], a member of the XPG superfamily of 5′ nucleases that includes EXO1, FEN1 and XPG [11], [12], [13]. In the second pathway, the nuclease making the initial cleavage of the junction is SLX1 in complex with SLX4 that binds a number of nucleases involved in DNA repair [14], [15], [16], [17], [18]. SLX1 is a member of the UvrC family of endonucleases and contains a GIY-YIG element that forms a metal ion-binding active center in a number of nucleases [19], [20]. However, unlike GEN1, SLX1 introduces a single cleavage into the junction; the unilaterally cleaved junction is then the substrate for the MUS81-EME1 nuclease that is also tethered to the SLX4 complex [21], [22]. Acting in isolation, the properties of MUS81-EME1 are akin to those of a flap endonuclease [23], [24], [25], but acting in concert with SLX1 generates a productive resolution. One or other of these two systems is required to be functional for cell viability; GEN1 and SLX4 are synthetically lethal in human cells as a result of unprocessed junctions leading to dysfunctional mitosis [26]. Ectopic expression of human GEN1 has been found to restore the meiotic phenotype of *mus81Δ* fission yeast [27], and Yen1 resolves persistent DNA junctions in meiotic yeast [28].

The N-terminal section of human GEN1 can act as a single protein in dimeric form to generate the productive resolution of a four-way junction [10]. It thus appears to have properties that are analogous to those of well-characterized junction-resolving enzymes from bacteria, phage, archaea and mitochondria [7]. However, more detailed structural and biophysical analysis of this protein has been hampered by some of its properties. In our hands, all fragments of human GEN1 tested have proved to be polydisperse and fail to form discrete complexes with junctions, and that behavior is evident in other published studies [10]. We therefore sought an ortholog with closely similar sequence and properties that was more suited to quantitative study. To that end, we investigated thermophilic fungi, as thermostable proteins often are well behaved in solution. We identified the GEN1 orthologs from a number of such species and expressed and purified GEN1 enzyme from *Chaetomium thermophilum*. We find that this protein is very well behaved in solution, binding to DNA junctions in dimeric form and generating bilateral cleavage by accelerating second-strand cleavage. Further investigation reveals that its properties are closely similar to those of non-eukaryotic resolving enzymes, and the enzyme is highly suitable for more detailed analysis.

## Results

### Identification of a gene encoding GEN1 in *C. thermophilum*

**Fig. 1:**
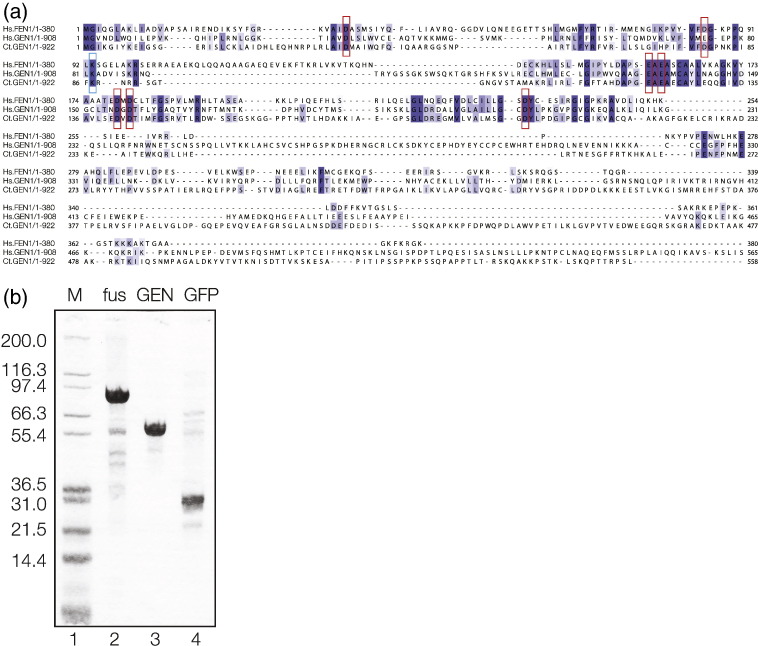
Identification and expression of a putative GEN1 sequence from *C. thermophilum*. (a ) Alignment of the protein sequences of human (Hs) FEN1 and GEN1 and the putative *C. thermophilum* (Ct) GEN1, shaded by identity. Seven conserved acidic amino acids involved in metal ion binding in the active site of FEN1 are boxed red, and the conserved lysine is boxed blue. (b) Expression of CtGEN1_1–487_. The protein fragment was expressed in *E. coli* as a fusion with GFP that was subsequently cleaved using TEV protease. Purified protein was analyzed by electrophoresis in 10% polyacrylamide containing SDS (Fisher). Tracks: 1, a mixture of proteins as a size marker, with molecular mass (kDa) written on the left; 2, the CtGEN1_1–487_-GFP fusion protein; 3, purified CtGEN1_1–487_; 4, GFP released from the fusion.

To identify the GEN1 gene in thermophilic fungi, we extended our previous phylogenetic analysis of XPG superfamily members that covered a set of FEN1, EXO1, XPG and GEN1-like sequences from phylogenetically diverse eukaryotes [9], and we included sequences from the thermophilic fungi *C. thermophilum, Myceliophthora thermophile*, *Talaromyces marneffei*, *Talaromyces stipitatus* and *Thielavia terrestris*. The full alignment is shown in Supplementary Fig. 1, and an unrooted phylogenetic tree is shown in Supplementary Fig. 2. The analysis confirms that XPG superfamily members from thermophilic fungi fall into four distinct classes represented by the XPG, FEN1, EXO1 and GEN1 nuclease families, allowing us to identify the putative GEN1 of *C. thermophilum*. An alignment of the N-terminal half of human FEN1 and GEN1 and the proposed *C. thermophilum* GEN1 is shown in Fig. 1a. Seven strongly conserved acidic amino acids are boxed red; these bind two active-site metal ions in the structure of human FEN1 [13] and are conserved in all the XPG superfamily of nucleases (Supplementary Fig. 1). In addition, lysine K87 (boxed blue) is conserved in all three proteins; this is close to the scissile phosphate in human FEN1 and probably stabilizes the anionic transition state of the hydrolysis reaction. The N-terminal 230 amino acids of the human and deduced *C. thermophilum* GEN1 sequences that include these conserved acidic residues are 26% identical.

### Bacterial expression of *C. thermophilum* GEN1

A synthetic gene encoding the N-terminal section of CtGEN1 comprising amino acids 1–487 optimized for *Escherichia coli* codon usage was inserted into the pET derivative pWaldo [29] and expressed in *E. coli* BL21(DE3) RIL. The translated protein that resulted from this was a fusion of CtGEN1_1–487_ with green fluorescent protein (GFP) at the C-terminus (the fluorescence of which could be monitored during purification) and an octahistidine tag at the C-terminus (Supplementary Fig. 3). The GFP could be cleaved from the fusion by virtue of an intervening tobacco etch virus (TEV) protease site. The protein was purified by sequential application to Ni^2 +^-charged metal affinity, heparin and gel-filtration columns. The purified protein migrated as a single band on electrophoresis in polyacrylamide in the presence of SDS (Fig. 1b) even when heavily overloaded (Supplementary Fig. 4). CtGEN1_1–487_ eluted from the gel-filtration column in between bovine serum albumin (BSA) (66 kDa) and carbonic anhydrase (29 kDa) (Supplementary Fig. 5), consistent with the protein existing in monomeric form (calculated molecular mass of 55.1 kDa). No elution corresponding to a dimer of CtGEN1_1–487_ was detected. Rass *et al.* similarly concluded that human GEN1_1–527_ is monomeric in free solution [10].

### *C. thermophilum* GEN1 is a nuclease that is selective for the structure of four-way DNA junctions

**Fig. 2:**
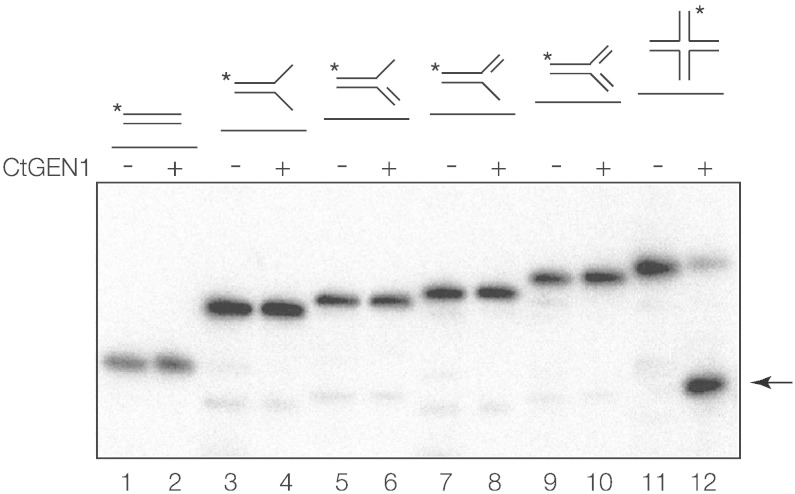
Substrate selectivity for CtGEN1_1–487_. A number of branched DNA species were prepared, with one strand radioactively 5′-^32^P-labeled (indicated by an asterisk). Each was incubated with (even tracks) or without (odd tracks) 50 nM CtGEN1_1–487_ in 10 mM Hepes (pH 7.5) in the presence of 10 mM MgCl_2_. Potential products were separated by electrophoresis in 6% polyacrylamide and visualized by phosphorimaging. The substrates were duplex (tracks 1 and 2), splayed duplex (tracks 3 and 4), 3′ flap (tracks 5 and 6), 5′ flap (tracks 7 and 8), nicked three-way junction (tracks 9 and 10) and four-way junction Jbm5 (tracks 11 and 12). Only the four-way junction was significantly cleaved, with the product arrowed.

Purified CtGEN1_1–487_ was assessed for nucleolytic activity on a variety of branched DNA substrates including various flap species, a nicked three-way junction and a four-way (4H) junction (Fig. 2 and Supplementary Fig. 6). The four-way junction used was Jbm5 [30] with a 12-bp core of homology that can consequently undergo 12 steps of branch migration. Each construct was incubated at 5 nM with 50 nM protein (single-turnover conditions) for 2 min at 25 °C and the resulting products were examined by gel electrophoresis. Under the conditions of the experiment, it is clear that there is strong cleavage of the four-way junction and very much weaker cleavage of the other species. Thus, CtGEN1_1–487_ is highly selective for the structure of the four-way DNA junction, consistent with a role as a junction-resolving enzyme.

### Mutation of conserved acidic amino acids leads to loss of cleavage of four-way DNA junctions

**Fig. 3:**
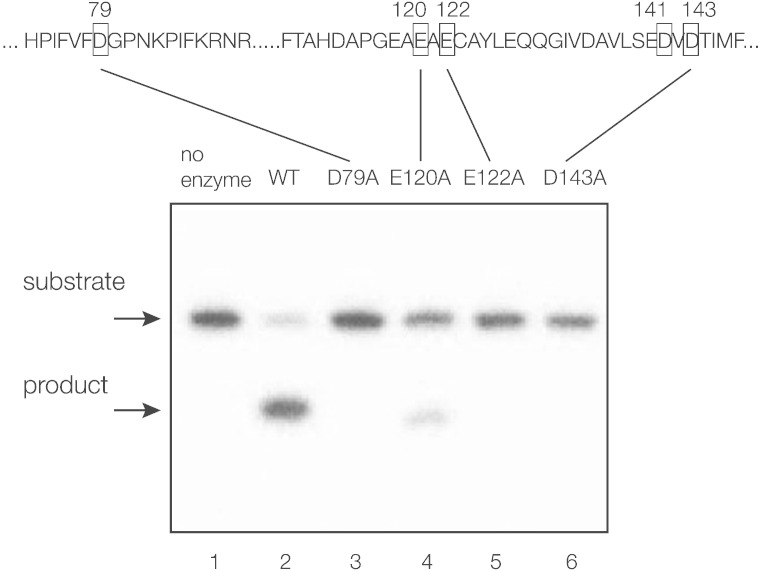
Cleavage activity on a DNA junction by putative active-site mutants of CtGEN1_1–487_. Conserved acidic residues were individually mutated to alanine and the cleavage activity against junction 3 was tested using purified CtGEN1_1–487_-GFP fusions. Junction 3 radioactively 5′-^32^P-labeled on the x strand was incubated without enzyme (track 1) or with wild type (track 2), D79A (track 3), E120A (track 4), E122A (track 5) or D143A (track 6) GEN1 for 4 min at 37 °C, and any products of cleavage were separated by electrophoresis in a 15% polyacrylamide and visualized by phosphorimaging.

The sequence alignment between CtGEN1 and human FEN1 (Fig. 1a) indicates a number of probable acidic amino acids likely to be involved in catalysis if these proteins are related, viz. D38, D79, E120, E122, E141, D143 and D199 (numbered for CtGEN1_1–487_). We have individually altered four of these by site-directed mutagenesis and tested the junction-cleaving activity in preparations of the proteins (as GFP fusions for preparative convenience). While the wild-type enzyme cleaves the DNA junction, those with D79A, E122A or D143A mutation lead to no cleavage of the junction (Fig. 3), even after prolonged incubation (data not shown). The equivalent carboxylate groups in human FEN1 are ~ 2.3 Å from the catalytic metal ions [13]. E120A has some activity though significantly lower than for the wild-type protein; in FEN1, this carboxylate is 4.8 Å from the metal ion, thus not directly bound. These data provide further evidence that the protein is indeed GEN1 of *C. thermophilum*.

### CtGEN1 introduces 2-fold symmetrical cleavages into a four-way DNA junction

**Fig. 4:**
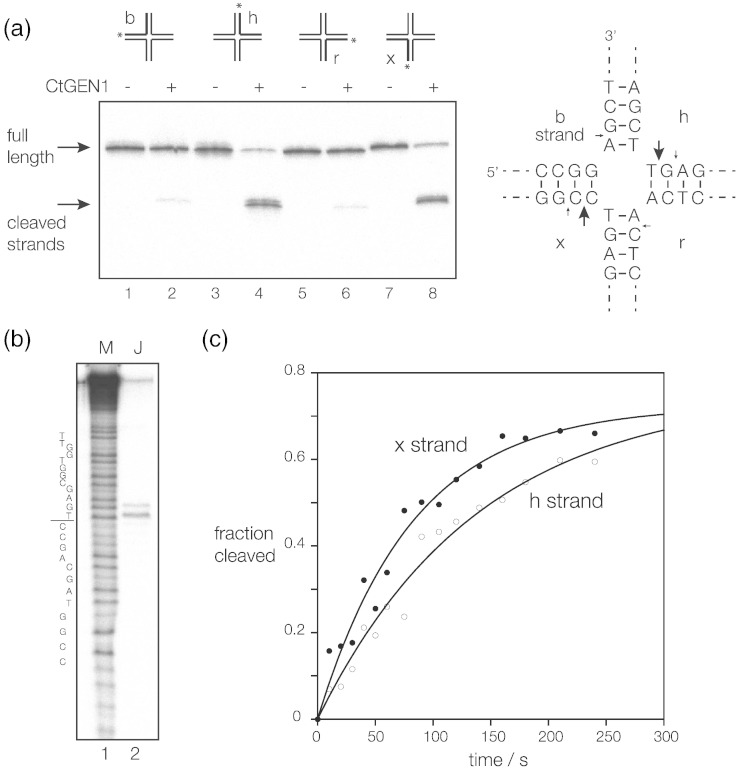
Location and rates of cleavage of the four strands of a four-way junction. Four versions of a junction with arms of 25 bp and a core sequence corresponding to junction 3 were each radioactively 5′-^32^P-labeled on a single strand. Each was incubated with 100 nM CtGEN1_1–487_ in 10 mM Hepes (pH 7.5), 10 mM MgCl_2_, 50 mM NaCl, 0.1% BSA and 1 mM DTT at 37 °C for 10 min, and the products were separated by electrophoresis in a 15% polyacrylamide and visualized by phosphorimaging. (a) Phosphorimage of the gel. Samples were applied in the order b, h, r and x strands labeled, with even- and odd-numbered tracks containing samples with and without enzyme added. All strands were cleaved to some degree, but the h and x strands were cleaved more strongly than the b or r strands. (b) The product of h-strand cleavage by CtGEN1_1–487_ was electrophoresed in a 15% polyacrylamide sequencing gel in TBE containing 8 M urea alongside a sequence ladder derived from partial chemical degradation of the h strand in order to map the positions of cleavage at nucleotide resolution. The sequence of the h strand is shown on the left, with the position of strand exchange indicated by the line. The positions of cleavage in all four strands are shown on the schematic of the central sequence of the junction, with the larger arrows indicating strong cleavage. (c) Reaction progress plotted as a function of time for the h (open circles) and x (filled circles) strands. The data have been fitted to single exponential functions (lines).

Productive resolution of a junction requires symmetrical cleavage of two strands; thus, we mapped the cleavages introduced into each strand of the well-characterized junction 3 [31] that is not capable of branch migration. The junction was prepared from four strands of 50 bp, generating a version of junction 3 having four arms each of 25 bp in length. Four radioactive forms were made, each individually 5′-^32^P-labeled on one strand. Each was incubated at a concentration of 1 nM with 50 nM CtGEN1_1–487_ for 10 min. The products were examined by gel electrophoresis under denaturing conditions and phosphorimaging (Fig. 4a). The h and x strands were strongly cleaved and the b and r strands were more weakly cleaved, such that the h and x strands were cleaved ≥ 20-fold greater than the b and r strands.

We compared the products of cleavage of junction 3 with those from an active N-terminal fragment of the human GEN1, HsGEN1_1–527_ (as the C-terminal fusion with GFP, i.e., HsGEN1_1–527_-GFP). In separate reactions, we incubated the same radioactive junctions with HsGEN1_1–527_-GFP and compared the products with those of CtGEN1_1–487_ (Supplementary Fig. 7). Both enzymes cleave at the same sites, with similar patterns of cleavage, in agreement with the sites mapped for human GEN1 by Rass *et al.* [10]. However, the difference between h, x and b, r cleavages for the human GEN1_1–527_ was less pronounced compared to the CtGEN1_1–487_ products.

Comparison of the position of migration of the CtGEN1_1–487_ product bands with those of a ladder generated from the same strand by chemical depurination shows that the major cleavage occurs 1 nt 3′ to the point of strand exchange (Fig. 4b). This is identical with the position of cleavage of a 5′ flap substrate by FEN1 and other members of that superfamily [32], [33], highlighting the similarity of the two enzymes.

We went on to measure the rates of cleavage of the four strands by CtGEN1_1–487_. The reaction was initiated by addition of 10 mM MgCl_2_ (final concentration), aliquots were removed at various times and the reaction terminated by denaturation in formamide and the products and substrate were separated by gel electrophoresis under denaturing conditions. The extent of cleavage at each time was quantified by phosphorimaging from which progress curves of fraction of cleavage as a function of time were plotted (Fig. 4c). The data were well fitted by single exponential functions, from which rates of cleavage of 0.008 and 0.01 s^− 1^ were measured for the h and x strands, respectively.

### CtGEN1 binds DNA junctions with high affinity

**Fig. 5:**
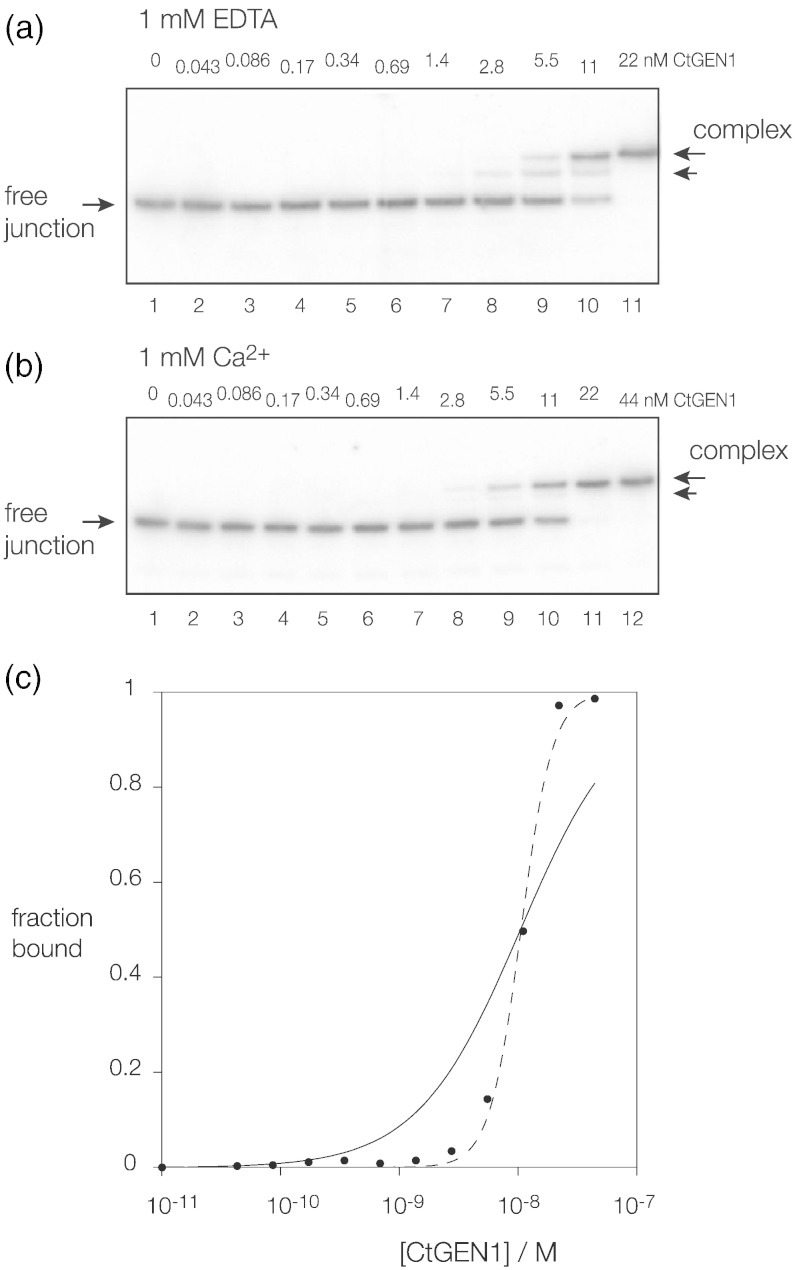
Binding of CtGEN1_1–487_ to junction 3. Junction 3 was prepared with 25 bp arms, radioactively 5′-^32^P-labeled on the x strand. We incubated 82 pM junction with increasing concentrations of CtGEN1_1–487_ in 10 mM Hepes (pH 7.5), 50 mM NaCl, 0.1% BSA and 1 mM DTT with either 1 mM EDTA or 1 mM Ca^2 +^ for 60 min and electrophoresed in 6% polyacrylamide under non-denaturing conditions. (a and b) Phosphorimages of the gels electrophoresed in (a) 1 mM EDTA and (b) 1 mM Ca^2 +^. Tracks contain DNA junction incubated with increasing concentrations of CtGEN1_1–487_ left to right. The concentration of CtGEN1_1–487_ is shown over each track. DNA–protein complexes are visible migrating more slowly than the free junction (arrowed right). A complex of intermediate mobility is visible in EDTA, but in Ca^2 +^, essentially only a single complex is evident. (c) The fractional intensity of the retarded complex in Ca^2 +^ is plotted as a function of CtGEN1_1–487_ concentration (filled circles). The data have been fitted to two models. A standard binding isotherm (line) gives an affinity *K*_d_ = 10 nM, but the data clearly exhibit cooperativity. They have therefore additionally been fitted to the Hill equation (broken line).

We have examined binding of CtGEN1_1–487_ to a four-way DNA junction using gel electrophoresis in polyacrylamide under non-denaturing conditions, as previously performed for junction-resolving enzymes of bacteriophage [34], [35], [36], mitochondrial [37], [38], [39], [40] and archaeal [41] origins. Radioactively 5′-^32^P-labeled junction 3 at 100 pM was incubated with a range of concentrations between 43 pM and 44 nM of CtGEN1_1–487_ and applied to a 6% polyacrylamide gel (Fig. 5a and b) and electrophoresed under non-denaturing conditions at room temperature. The experiment was performed under two different conditions, in the presence of either 1 mM ethylenediaminetetraacetic acid (EDTA) (to chelate trace metal ions) or 1 mM Ca^2 +^. CtGEN1_1–487_ is catalytically inactive in Ca^2 +^; thus, in general, we employ this cation in binding experiments. Slower-migrating species were observed with the addition of increasing concentrations of protein in both cases, indicative of complex formation. However, in EDTA, a species of intermediate mobility was observed, whereas in Ca^2+^, the complex migrated largely as a single retarded species. It is likely that the intermediate species observed in the presence of EDTA is a complex with a single monomer of CtGEN1_1–487_ bound to the junction, while the slower species is a complex with a dimer of CtGEN1_1–487_ (see the following section). The fraction of bound junction was estimated by quantification of bound and unbound species and plotted as a function of protein concentration (Fig. 5c). The isotherms were fitted to a simple binding model, yielding an estimate of the affinity of the GEN1 fragment for a DNA four-way junction of *K*_d_ = 10 nM. However, it is clear that the binding is more cooperative than a simple two-state binding process, as previously observed for RuvC [42], Ydc2 [39] and Hjc [41] junction-resolving enzymes. Fitting the data to the standard Hill equation gives a significantly better fit, consistent with a strongly cooperative binding of CtGEN1_1–487_ as a dimeric complex the presence of Ca^2 +^.

### CtGEN1 binds to DNA junctions as a dimer

**Fig. 6:**
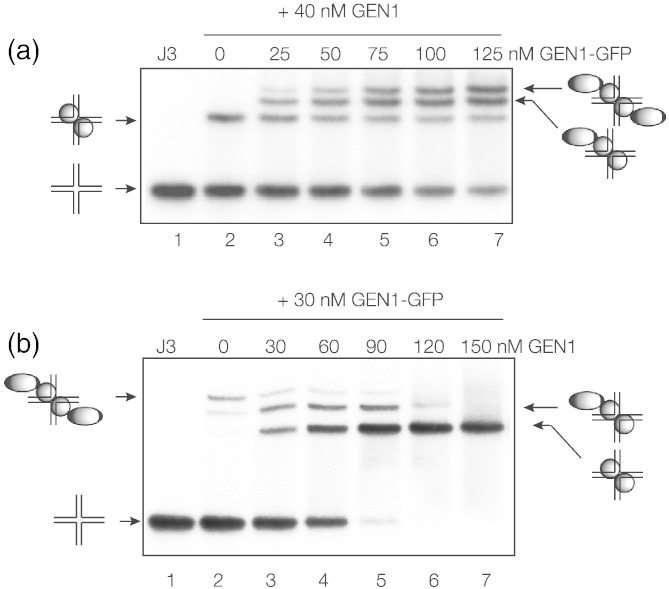
CtGEN1_1–487_ binds to a DNA junction as dimer. Complexes have been formed with CtGEN1_1–487_ alone and fused to GFP. These have been analyzed by gel electrophoresis in 6% polyacrylamide. In each case, free junction was electrophoresed in track 1. The experiment was performed in two ways. (a) We incubated 160 nM junction with 40 nM CtGEN1_1–487_ in 10 mM Hepes (pH 7.5), 50 mM NaCl, 0.1% BSA, 1 mM DTT and 1 mM CaCl_2_ (track 2), with increasing concentrations of CtGEN1-GFP fusion (tracks 3–7; concentrations indicated above each track). (b) We incubated 80 nM junction with 40 nM CtGEN1-GFP fusion in 10 mM Hepes (pH 7.5), 50 mM NaCl, 0.1% BSA, 1 mM DTT and 1 mM CaCl_2_ (track 2), with increasing concentrations of CtGEN1_1–487_ fusion (tracks 3–7; concentrations indicated above each track). Note that, in each case, three retarded complexes are observed, consistent with CtGEN1_1–487_ binding as a dimer to the DNA junction.

The results in the previous section suggest that CtGEN1_1–487_ binds as a dimer, but we sought a more conclusive test of this. We have shown previously for other junction-resolving enzymes that, provided subunit exchange is possible, electrophoretic examination of complexes using mixtures of the enzyme and longer versions generated by fusion with another protein domain together can provide information on the oligomeric state of the protein in the complex [35], [36], [37], [39], [43]. For these experiments, we used the C-terminal fusion of GFP with CtGEN1_1–487_. The experiment was performed in two ways. First, we incubated radioactively labeled junction 3 with 40 nM CtGEN1_1–487_ (i.e., the non-fusion) and made five further equivalent incubations with addition of the CtGEN1_1–487_-GFP fusion at individual concentrations between 25 and 125 nM (Fig. 6a). Incubation with CtGEN1_1–487_ resulted in a single retarded band of junction–protein complex. However, addition of the CtGEN1_1–487_-GFP led to three complex bands, with the relative intensity of the slowest increasing with the molar fraction of the fusion protein. This indicates that the protein binds as a dimer to the junction, thereby generating complexes with three combinations of the two proteins, that is, non-fusion + non-fusion, non-fusion + fusion and fusion + fusion, with fastest, intermediate and slowest mobilities, respectively.

The experiment was also carried out with a fixed 30 nM concentration of CtGEN1_1–487_-GFP fusion and addition of increasing concentrations (30–180 nM) of CtGEN1_1–487_ (Fig. 6b). Once again, three species of different mobility were observed with equimolar CtGEN1_1–487_ and CtGEN1_1–487_-GFP, but as the mole fraction of CtGEN1_1–487_ increased, only the fastest species was observed. This confirms the dimeric nature of the protein in the complex. It is furthermore apparent from these data that the C-terminal fusion of GFP lowers the affinity of CtGEN1_1–487_ for the four-way junction.

### CtGEN1 exhibits acceleration of second-strand cleavage

A resolving enzyme must introduce bilateral cleavage within the lifetime of the protein–DNA complex in order to generate complete resolution of a four-way junction. We have previously used the cleavage of a supercoil-stabilized cruciform structure to demonstrate bilateral cleavage by phage, bacterial and yeast mitochondrial resolving enzymes [36], [44], [45], [46], [47]. The cruciform structure is intrinsically unstable except in a negatively supercoiled circular DNA [48] and is rapidly reabsorbed if the covalent continuity of the circle is broken at any point. If the enzyme makes a single cleavage in the DNA, the cruciform can only remain extruded if its integrity is preserved by the DNA–protein interactions in the complex. If the protein dissociates before second-strand cleavage occurs, the cruciform is reabsorbed and no substrate remains for a second cleavage reaction. We have applied this approach to the analysis of junction cleavage by CtGEN1_1–487_.

**Fig. 7:**
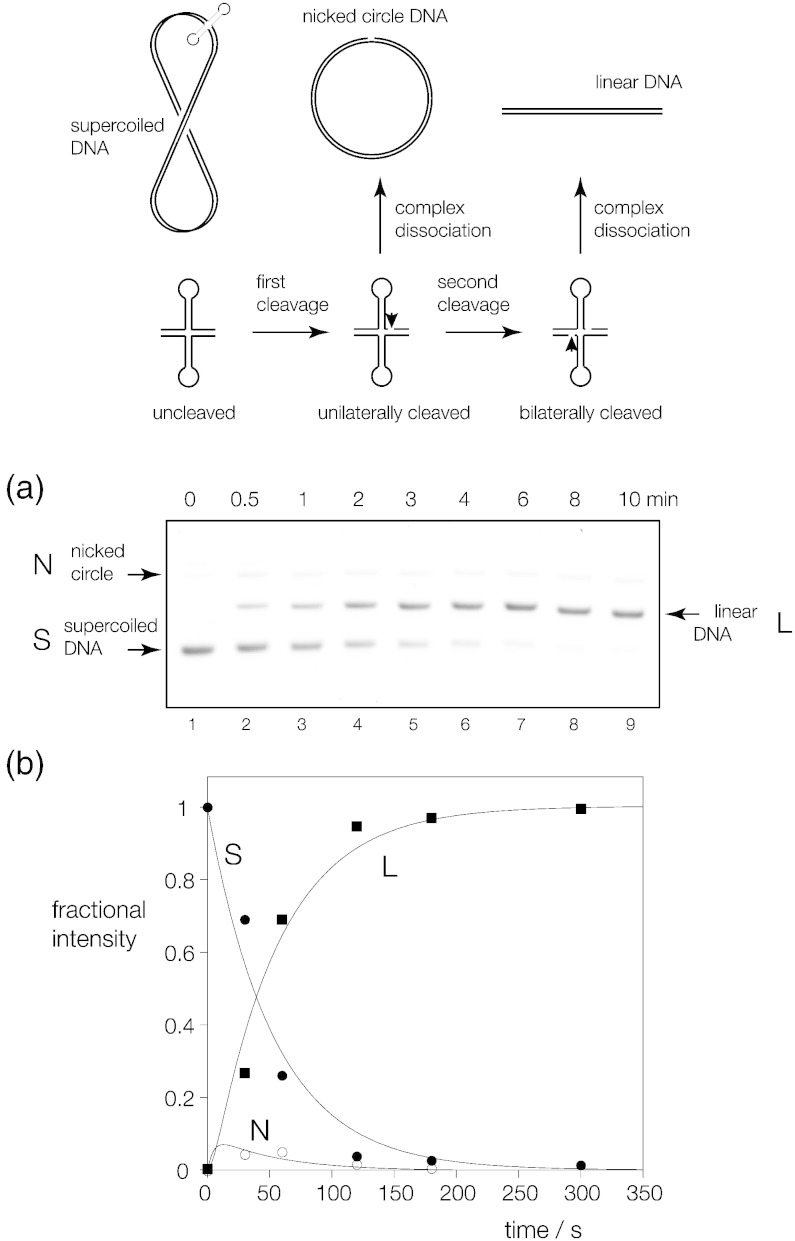
Analysis of bilateral cleavage in a DNA junction using a supercoil-stabilized cruciform substrate. The principle of the experiment is shown in the scheme (top). A cruciform structure contains a four-way junction that is a substrate for resolving enzymes. However, the cruciform requires negative supercoiling for stabilization. Unilateral cleavage of the junction followed by dissociation of the protein leads to the formation of a nicked circle in which the cruciform substrate is no longer present. By contrast, subsequent second cleavage within the lifetime of the complex generates a linear product as the cruciform is now bilaterally cleaved. (a) The supercoiled DNA substrate and the nicked and linear products are readily separated by electrophoresis in 1% agarose. Supercoiled plasmid pBHR3 was incubated with 200 nM CtGEN1_1–487_ in 10 mM Hepes (pH 7.5), 50 mM NaCl, 1 mM DTT and 0.1% BSA at 37 °C. After the cleavage reaction was initiated by addition of MgCl_2_ to 10 mM, samples were removed at different times, protein was removed by treatment with proteinase K and electrophoresed in a 1% agarose gel and DNA was fluorescently stained. A fluoroimage is shown. With time, the supercoiled DNA is converted to linear product, with a low intensity of nicked circular DNA appearing as a transient intermediate. (b) The intensities of the three species were quantified and plotted as a function of time (lower). These data are fitted to the integrated rate Eqs. (3), (4), (5) shown in the main text.

For this purpose, we used a 34-bp cruciform that is stably extruded in the plasmid pBHR3 (Supplementary Fig. 8) extracted from *E. coli* in exponential growth (Fig. 7). Supercoiled pBHR3 was incubated CtGEN1_1–487_ and aliquots were removed at different times. The topological state of the plasmid was then examined by gel electrophoresis in 1% agarose (Fig. 7a). Uncleaved supercoiled plasmid migrates as the fastest species. Any unilateral cleavage by the enzyme converts the plasmid into a nicked circle, which migrates slowly. By contrast, bilateral cleavage generates a linear DNA product of intermediate mobility.

Incubation of pBHR3 with CtGEN1_1–487_ results in loss of supercoiled DNA and formation of linear product, indicative of bilateral cleavage occurring within the lifetime of the enzyme–junction complex. However, a smaller fraction of nicked circular DNA is also visible at early times. The intensities of the different species have been quantified and plotted as a function of time (Fig. 7b). This shows the conversion of supercoiled to linear DNA product over the time course. Nicked circular DNA appears as a transient intermediate, being formed at early times and subsequently being converted to linear product. The cruciform substrate would be unstable in an unconstrained nicked circle; thus, it must be preserved within the bound complex with the resolving enzyme.

We have analyzed these data in terms of a kinetic model, whereby the first cleavage occurs at a rate *k*_1_, followed by cleavage of the other strand with rate *k*_2_. In this scheme, we make no attempt to distinguish the identity of the strand that is cleaved first. Thus, the rates of change of supercoiled DNA (concentration [S]*_t_* at time *t*) and nicked circular DNA ([N]*_t_* at time *t*) will be given by:

(1) $$d\left[S\right]/dt=-k_{1}{\left[S\right]}_{t}$$

(2) $$d\left[N\right]/dt=k_{1}{\left[S\right]}_{t}-k_{2}{\left[N\right]}_{t}$$

Integration of these equations by standard analytical methods leads to the kinetic rate equations expressing the instantaneous fractional concentrations of the three species as:

(3) $${\left[S\right]}_{t}/\;{\left[S\right]}_{0}=\exp\left(-k_{1}t\right)$$

(4) $${\left[N\right]}_{t}/\;{\left[S\right]}_{0}=k_{1}/\left(k_{2}-k_{1}\right)\cdot \left(\,\exp\left(-k_{1}t\right)-\exp\left(-k_{2}t\right)\right)$$

and by conservation, the fractional concentration of linear species [L]*_t_* at time *t* will be:

(5) $${\left[L\right]}_{t}/{\left[S\right]}_{0}=1-\exp\left(-k_{1}t\right)-k_{1}/\left(k_{2}-k_{1}\right)\cdot \left(\,\exp\left(-k_{1}t\right)-\exp\left(-k_{2}t\right)\right)$$

The measured intensities of the three species have been fitted to these equations (Fig. 7b), giving the rates *k*_1_ = 0.019 and *k*_2_ = 0.20 min^− 1^. From these rates, we see that the rate of cleavage of the nicked species is 11-fold greater than that of the supercoiled DNA; that is, there is an acceleration of second-strand cleavage relative to the first. The effect of this is to ensure that bilateral cleavage occurs within the lifetime of the enzyme–junction complex.

## Discussion

We have identified the ortholog of GEN1 from the thermophilic fungus *C. thermophilum*. The sequence aligns with human GEN1 and with the putative genes for the other XPG superfamily of 5′ nucleases EXO1, FEN1 and XPG. The alignments identify the probable active-site residues, some of which we have confirmed by mutagenesis. Expression of a fragment of the CtGEN1 protein reveals that it possesses all the properties expected of a junction-resolving enzyme.

CtGEN1_1–487_ exhibits a strong selectivity for the structure of the four-way DNA junction and cleaves with a rate of ~ 0.5 min^− 1^. The strands are cleaved predominantly 1 nt 3′ to the point of strand exchange, analogous to the positions of cleavage of 5′ flap structures by FEN1 [32], [33], further strengthening assignment of the CtGEN1 gene. CtGEN1_1–487_ binds to DNA junctions with nanomolar affinity to form a discrete complex. Use of C-terminal protein fusions shows that the bound form is a dimer (although monomeric in free solution), consistent with the observed cleavage pattern. In the absence of added metal ions, some binding in monomeric form is observed at intermediate protein concentrations, but in the presence of Ca^2 +^, the complex contains a dimer of the protein, consistent with the requirement for two spatially separated cleavages to generate a productive resolution of the junction. Under the latter, more physiologically relevant conditions, binding occurs with strong cooperativity. Evidently, the C-terminal section of CtGEN1 is not required for the binding in dimeric form, although we note that C-terminal fusion with GFP substantially weakens the affinity, and thus, in principle, the deleted section of the protein might modulate the dimerization.

CtGEN1_1–487_ introduces bilateral cleavage into the junction within the lifetime of the enzyme–junction complex. Kinetic analysis of supercoiled cruciform cleavage shows that the rate of second-strand cleavage is greater than that of the first by a factor of 11. Acceleration of the second-strand cleavage increases the probability that both cleavages occur before dissociation of the bound complex and thus provides a kinetic mechanism that will tend to ensure a productive resolution of the junction. The yeast mitochondrial enzyme Cce1 exhibits closely similar behavior, with a 10-fold acceleration of second-strand cleavage [46].

The two sets of cleavages observed with junction 3 are consistent with the formation of alternative 2-fold symmetrical complexes in which the h and x or the b and r strands are cleaved, with the former predominating over the latter by a ratio of ~ 25:1. This pattern of cleavage (i.e., cleavage intensity b = r ≠ h = x) is commonly observed in other well-studied junction-resolving enzymes, where a dimeric protein binds to a potentially 4-fold symmetrical junction lowering the symmetry to a single dyad axis.

All these properties are closely similar to those of the well-characterized junction-resolving enzymes of bacteriophage [34], [35], [36], [45], bacterial [47], [49], mitochondrial [37], [38], [39], [40], [46] and archaeal [30], [41], [50] origins. Thus, eukaryotic GEN1 seems to be a canonical junction-resolving enzyme in all its properties analyzed to date. This is in marked contrast to the alternative pathway, involving the complex SLX1-SLX4-MUS81-EME1, requiring at least four different proteins and two nuclease activities to generate the two cleavages essential for resolution [21], [22].

As frequently observed with proteins derived from thermophilic organisms, CtGEN1_1–487_ is very well behaved in solution. Of particular note, this protein is free of the aggregation properties that have hindered the analysis of the human protein. Whereas the human protein typically binds to DNA junctions to generate multiple different complexes (found both by Rass *et al*. [10] and in our own unpublished observations), CtGEN1_1–487_ binding results in the formation of a discrete complex with a DNA junction. This makes this an excellent subject for quantitative biophysical, structural and mechanistic studies. We are presently performing crystallization trials with the enzyme and have obtained crystals of a complex of CtGEN1_1–487_ bound to a DNA junction that are in the process of optimization.

## Materials and Methods

### Bioinformatic analysis of XPG family proteins

XPG superfamily sequences were aligned using JALVIEW employing the MAFT algorithm [51]. An unrooted phylogenetic tree was generated using the Splitstree program [52] and employing the neighbor joining method.

### Cloning and expression of a gene encoding CtGEN1

A synthetic gene expressing CtGEN1_1–487_ was obtained from GeneArt (Life Technologies) and cloned into pWaldo, a variant of the pET28 [29]. This generates CtGEN1_1–487_ with GFP at its C-terminus linked via a site for TEV protease and with an octahistidine C-terminal peptide to facilitate purification (Supplementary Fig. 3). Plasmids were transformed into BL21(DE3) RIL immediately before cell preparation and were grown in Luria Broth medium supplemented with kanamycin and chloramphenicol. Cells were initially grown at 37 °C until the absorbance at 600 nm (*A*_600_) of the cell culture reached 0.4 whereupon the cells were transferred to 20 °C. Once the culture reached *A*_600_ = 0.6, expression of CtGEN1_1–487_ was induced by addition of 0.1 mM IPTG and incubated for a further ~ 20 h. Cells were harvested by centrifugation at 5000*g*, resuspended (at 3 ml/g of cell paste) on ice and lysed in 50 mM NaH_2_PO_4_ (pH 8.0), 1 M NaCl, 1 mg/ml lysozyme and 0.2% Triton X100. The cellular extract was sonicated and clarified by centrifugation at 20,000*g* for 30 min at 4 °C. The extract was then applied to Ni^2 +^-IDA metal chelate resin (Generon) and eluted in 50 mM NaH_2_PO_4_ (pH 8.0) and 1 M NaCl with a gradient of 0–0.5 M imidazole. Elution was monitored by the green color of GFP, and CtGEN1_1–487_ eluted around 150 mM imidazole. The protein was dialyzed against 25 mM Hepes (pH 7.5), 0.1 M NaCl and 1 mM DTT, and 1/10 (mol/mol) of TEV protease was added to the protein extract. The protein extract was applied to a Hitrap Heparin HP column (GE healthcare) and eluted in a gradient of 0.1–1 M NaCl in a background of 25 mM Hepes (pH 7.5). GFP does not bind to the heparin column under these conditions and CtGEN1_1–487_ eluted at ~ 0.4 M NaCl. The protein was then subjected to gel filtration by application to a Superose 12 column in 25 mM Hepes (pH 7.5) and 1 M NaCl. The peak fraction of CtGEN1_1–487_ was dialyzed against 25 mM Hepes (pH 7.5), 0.1 M NaCl and 50% glycerol and was stored at − 80 °C. The concentration of CtGEN1_1–487_ was estimated by absorbance at 280 nm with an estimated *A*_280_ = 50,600 M^− 1^ cm^− 1^ (per monomer). Protein was analyzed by electrophoresis in 10% EZ-RUN™ SDS polyacrylamide gels (Fisher Scientific) following the manufacturer's instructions at 200 V for 50 min. Gels were stained using Quick Coomassie stain (Generon) and destained in water.

### Synthesis of DNA oligonucleotides and construction of junction species

Oligonucleotides were synthesized using β-cyanoethyl phosphoramidite chemistry. Fully deprotected oligonucleotides were purified by gel electrophoresis in polyacrylamide gels [10–20% (w/v) depending upon oligonucleotide length] in 90 mM Tris–borate (pH 8.5) and 2 mM EDTA (TBE buffer) containing 8 M urea and were recovered by electroelution and ethanol precipitation. Helical junctions were assembled by mixing stoichiometric quantities of strands (all sequences shown in Supplementary Fig. 6) and were annealed by incubation in 20 mM Tris–HCl (pH 8) and 50 mM NaCl for 5 min at 85 °C, followed by slow cooling. These were purified by electrophoresis under non-denaturing conditions in 8% polyacrylamide in TBE buffer for 8 h at 20 °C and were recovered by electroelution and ethanol precipitation.

### Analysis of cleavage of branched DNA species by CtGEN1

A variety of flap-containing and branched DNA species were tested for cleavage by CtGEN1_1–487_. We incubated 50 nM CtGEN1_1–487_ with 5–10 nM DNA radioactively 5′-^32^P-labeled on one strand in 10 mM Hepes (pH 7.5), 10 mM MgCl_2_, 50 mM NaCl, 0.1 mg/ml BSA and 1 mM DTT for 1 h. Any reaction was terminated by addition of 50 mM EDTA and 1 μg/ml proteinase K (final concentrations). Substrates and products separated by electrophoresis in 6% (29:1) polyacrylamide in TBE buffer at 120 V for 4 h. Gels were dried and exposed to storage phosphor screens, imaged using a Fuji BAS 1500 phosphorimager and quantified using MacBAS software.

### Mapping the cleavage site on junction 3 by CtGEN1

The product of cleavage of junction 3 radioactively 5′-^32^P-labeled on one strand was analyzed by electrophoresis alongside the same strand after depurination. This was performed by incubation in 10 μl of 50 mM sodium citrate (pH 4) and 0.1 mM EDTA for 15 min at 80 °C as described by Wang *et al.* [53]. The strand was then cleaved at depurinated positions by addition of 115 μl of water and 15 μl of 10 M piperidine followed by incubation at 90 °C for a further 30 min. DNA was precipitated with isopropanol followed by NaCl and ethanol. Electrophoresis was performed using 15% sequencing gels in TBE buffer containing 8 M urea.

### Analysis of cleavage a four-way DNA junction using point mutants of CtGEN1

D79, E120, E122 and D143 were individually converted into alanine into CtGEN1_1–487_ by PCR of the gene using the Q5 site-directed mutagenesis kit (BioLab) following the manufacturer's instructions. Mutations were verified by DNA sequencing. Mutant CtGEN1_1–487_ was expressed and purified as a fusion with GFP as described above, except that the incubation with TEV protease and gel-filtration steps were omitted. Wild type and mutant CtGEN1_1–487_-GFP eluted in a similar manner from the heparin column. Protein concentration was estimated by absorbance at 280 nm using *A*_280_ = 72,550 M^− 1^ cm^− 1^ (per monomer). Analysis of junction cleavage was performed as described below except that junction was incubated with 200 nM CtGEN1_1–487_-GFP in the presence of 10 μg/ml of calf thymus DNA for 4 min at 37 °C.

### Analysis of cleavage kinetics of a four-way DNA junction by CtGEN1

Cleavage kinetics were analyzed under single-turnover conditions. We incubated 50–100 nM CtGEN1_1–487_ with 5–10 nM junction 3 radioactively 5′-^32^P-labeled on one strand in 10 mM Hepes (pH 7.5), 50 mM NaCl, 0.1% BSA and 1 mM DTT for 1 h. Cleavage was initiated by addition of 10 mM MgCl_2_ and aliquots were removed at chosen times and EDTA was added to 50 mM final concentration to terminate the reaction. One volume of 90% formamide was added and the samples were then denatured at 90 °C for 15 min. before separation of substrates and products by electrophoresis in a 15% (19:1) polyacrylamide gel in TBE containing 8 M urea at 80 W. Gels were dried and exposed to storage phosphor screens and quantified using a Fuji BAS 1500 phosphorimager using MacBAS software. The fraction of DNA cleaved at time *t* (*F_t_*) was fitted by nonlinear regression analysis to the equation:

(6) $$F_{t}=F_{f}.\left(1-\exp\left(-k_{c}t\right)\right)$$

where *F*_f_ is the fraction of DNA cleaved at the end of the reaction and *k*_c_ is the rate of cleavage.

### Cleavage of cruciform substrates in supercoiled DNA

The cruciform-containing plasmid pBHR3 was generated by ligating the oligonucleotides 5′ AATTCAAGGGGCTGTATATATATATATATATATATATACAGCCTGAGCG and 5′ GATCCGCTCAGGCTGTATATATATATATATATATATATACAGCCCCTTG between the *EcoRI and BamHI* sites of pAT153 (Supplementary Fig. 8). The central alternating adenine-thymine repeated sequence ensures facile extrusion of the cruciform in negatively supercoiled DNA [54]. This was transformed into *E. coli* DH5α and grown to *A*_600_ = 0.6 at 37 °C. The plasmid was amplified by treatment with 150 μg/ml chloramphenicol overnight. Plasmid DNA was isolated using Qiagen maxiprep purification kit at 4 °C and purified by two rounds of isopycnic CsCl/ethidium bromide ultracentrifugation. Supercoiled DNA was recovered by side puncture, ethidium bromide was removed by extraction with *n*-butanol and the DNA was subjected to extensive dialysis against 20 mM Tris (pH 8.0) and 1 mM EDTA to remove CsCl. DNA concentration was measured spectrophotometrically.

We preincubated 10 nM pBHR3 plasmid with 200 nM CtGEN1 in 10 mM Hepes (pH 7.5), 50 mM NaCl, 1 mM DTT and 0.1% BSA for 3 min at 37 °C before the cleavage reaction was initiated by addition of MgCl_2_ to a final concentration 10 mM. Aliquots were removed at specific times and 50 mM EDTA was added to terminate the reaction. We added 1 μl proteinase K and we incubated the samples overnight at room temperature. Supercoiled, linear and nicked DNAs were separated by electrophoresis in 1% agarose gels in TBE buffer. Gels were stained with Safeview nucleic acid stain (NBS Biological Ltd), extensively washed with water and scanned using a FLA-2000 fluorescent image analyzer (Fuji) using excitation at 473 nm with an emission filter at 580 nm.

### Analysis of binding of CtGEN1 to DNA four-way junctions

Radioactively-labelled J3 (at concentrations given in the text) was incubated with increasing concentrations of CtGEN1_1–487_ in 10 mM Hepes (pH 7.5), 50 mM NaCl, 0.1% BSA and 1 mM DTT with either 1 mM EDTA or 1 mM Ca^2 +^ for 1 h at 20 °C. After the addition of Ficoll-400 to 2.5%, the samples were immediately loaded onto a 6% (37:1) polyacrylamide gel in TBE and subjected to electrophoresis for 4 h. Gels were dried onto Whatman 3MM paper and analyzed by phosphorimaging as described above. Data were analyzed as fraction DNA bound (*f*_b_) *versus* protein concentration and fitted by nonlinear regression analysis to the equation:

(7) $$f_{b}=\left\{K_{d}+P_{t}+D_{t}–{\left({\left(K_{d}+P_{t}+D_{t}\right)}^{2}–4P_{t}\cdot D_{t}\right)}^{1/2}\right\}/2D_{t}$$

where *K*_d_ is the dissociation constant, *P*_t_ is the total protein dimer concentration and *D*_t_ is the total DNA concentration. The data were also fitted to the Hill equation for cooperative binding:

(8) $$f_{b}={P_{t}}^{n}/\left({P_{t}}^{n}+K_{d}\right)$$

where *n* is the Hill coefficient.
