# Supplementary material for: GEN1 from a Thermophilic Fungus Is Functionally Closely Similar to Non-Eukaryotic Junction-Resolving Enzymes
Source: J Mol Biol. 2014 Dec 12;426(24):3946–59. doi: 10.1016/j.jmb.2014.10.008 (PMC4270448; doi:10.1016/j.jmb.2014.10.008)
Supplement: Supplementary file 1 — Supplementary material. [file mmc1.pdf]

The functional properties of GEN1 from a thermophilic fungus are closely similar to non-eukaryotic junction-resolving enzymes

A. D. J. Freeman, Y. Liu, A.-C. Déclais, A. Gartner and D. M. J. Lilley

SUPPLEMENTARY INFORMATION

Supplementary Figures

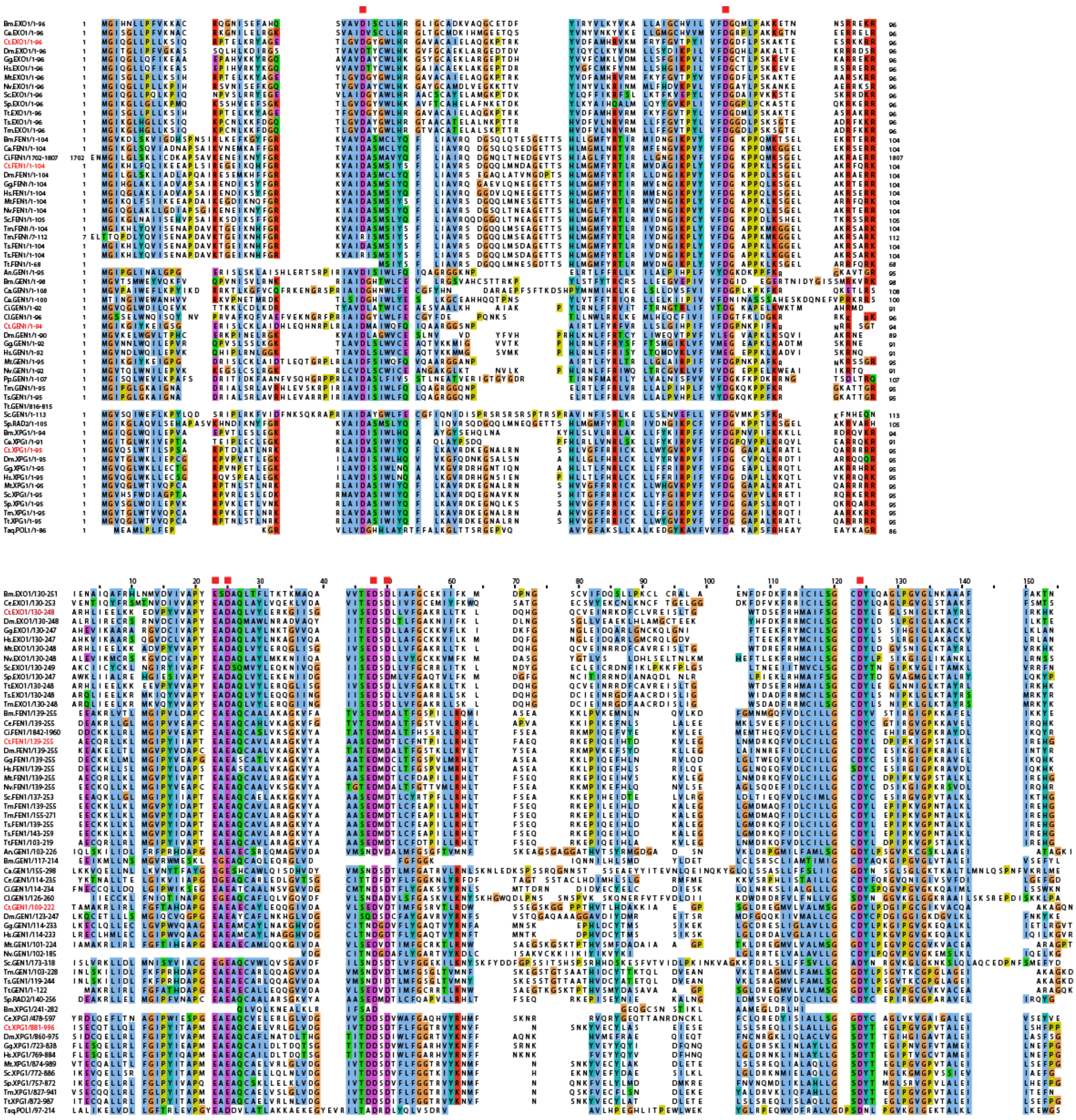

Supplementary Figure 1. Alignment of sections of orthologs of the XPG superfamily from a variety of organisms, colored by amino acid type. The conserved acidic amino acids are indicated by the red boxes above the columns. The *C. thermophilum* sequence names are highlighted red. An, *Aspergillus nidulans*, Bm, *Brugia malayi*, Ca, *Candida albicans*, Ci, *Ciona intestinalis*, Kl. *Kluyveromyces lactis*, Ct, *Chaetomium thermophilum,* Dm, *Drosophila melanogaster*, Gg, *Gallus gallus*, Hs, *Homo sapiens*, Mt, *Myceliophthora thermophile*, Nv, *Nematostella vectensis*, Pp, *Pichia pasteuris*, Sc, *Saccharomyces cerevisiae*, Sp, *Schizosaccharomyces pombe* Tm, *Talaromyces marneffei*, Ts, *Talaromyces stipitatus*, Tt, *Thielavia terrestris*. Bacterial *Taq*. Pol1 serves as an outgroup.

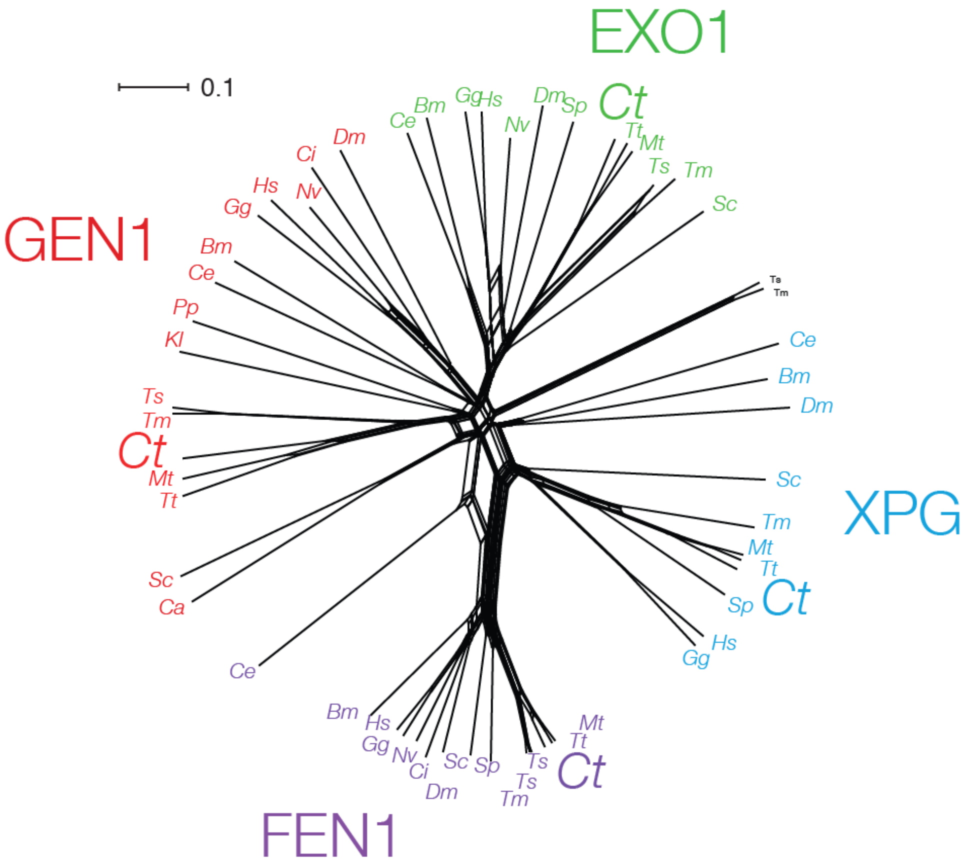

Supplementary Figure 2. Four distinct XPG family members occur in all animals examined. An unrooted phylogenetic tree of XPG-superfamily members is shown. The scale bar indicates the length of the branches of the phylogenetic trees corresponding to a 10% chance (p 0.1) of replacing an amino acid/site. An, *Aspergillus nidulans*, Bm, *Brugia malayi*, Ca, *Candida albicans*, Ci, *Ciona intestinalis*, Kl. *Kluyveromyces lactis*, Ct, *Chaetomium thermophilum,* Dm, *Drosophila melanogaster*, Gg, *Gallus gallus*, Hs, *Homo sapiens*, Mt, *Myceliophthora thermophile*, Nv, *Nematostella vectensis*, Pp, *Pichia pasteuris*, Sc, *Saccharomyces cerevisiae*, Sp, *Schizosaccharomyces pombe* Tm, *Talaromyces marneffei*, Ts, *Talaromyces stipitatus*, Tt, *Thielavia terrestris*.

MGIKGIYKEIGSGERISLCKLAIDHLEQHNRPLRLAIDMAIWQFQIQAARGGSNPAIRTLFYRFV

RLLSLGIHPIFVFDGPNKPIFKRNRRSGTGNGVSTAMAKRLIRLFGFTAHDAPGEAEAECAYLE

QQGIVDAVLSEDVDTIMFGSRVTLRDWSSEGSKGGPPTHVTLHDAKKIAEGPSGLDREGMVLV

ALMSGGDYLPDGIPGCGIKVACQAAKAGFGKELCRIKRADKEAITEWKQRLLHELRTNESGFF

RTKHKALEIPENFPNMEVLRYYTHPVVSSPATIERLRQEFPPSSTVDIAGLREFTRETFDWTFRP

GAIKLIKVLAPGLLVQRCLDRYVSGPRIDDPDLKKKEESTLVKGISMRREHFSTDATPELRVSFI

PAELVGLDPGQEPEVQVEAFGRSGLALNSDDEFDEDISSSQKAPKKPFDPWQPDLAWVPETILK

LGVPVTVEDWEEGQRSKGRAKEDKTAAKAKRKTKIIQSGSENLYFQGQFSKGEELFTGVVPIL

VELDGDVNGHKFSVSGEGEGDATYGKLTLKFICTTGKLPVPWPTLVTTLTYGVQCFSRYPDH

MKRHDFFKSAMPEGYVQERTISFKDDGNYKTRAEVKFEGDTLVNRIELKGIDFKEDGNILGHK

LEYNYNSHNVYITADKQKNGIKANFKIRHNIEDGSVQLADHYQQNTPIGDGPVLLPDNHYLST

QSALSKDPNEKRDHMVLLEFVTAAGITHGMDELYKKLAAHHHHHHHH

Supplementary Figure 3. The complete sequence of the CtGEN-$1_{1\text{-}487}$-TEV-GFP fusion protein expressed in *E. coli*. The CtGEN-$1_{1\text{-}487}$ protein is colored blue, and the GFP green. The intervening TEV protease site is colored cyan and the terminal octa-histidine peptide used in the purification red.

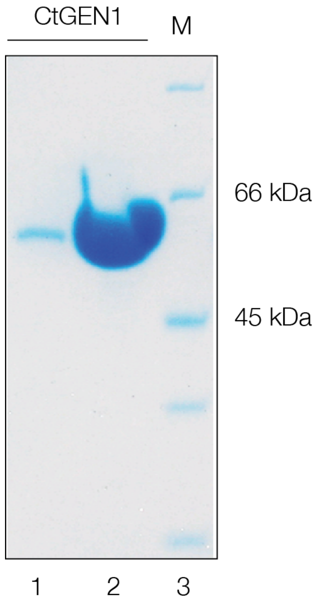

Supplementary Figure 4. Purified $CtGEN1_{1\text{-}487}$ expressed in *E. coli* analysed by gel electrophoresis in polyacrylamide in the presence of SDS. Tracks 1 and 2 contain purified $CtGEN1_{1\text{-}487}$ at different loadings; that in track 2 is heavily overloaded in an effort to reveal impurities. Track 3 contains a mixture of proteins to act as size markers. The calculated molecular mass of $CtGEN1_{1\text{-}487}$ is 55.1 kDa. Note that $CtGEN1_{1\text{-}487}$ migrates as a single species, with no other polypeptides visible.

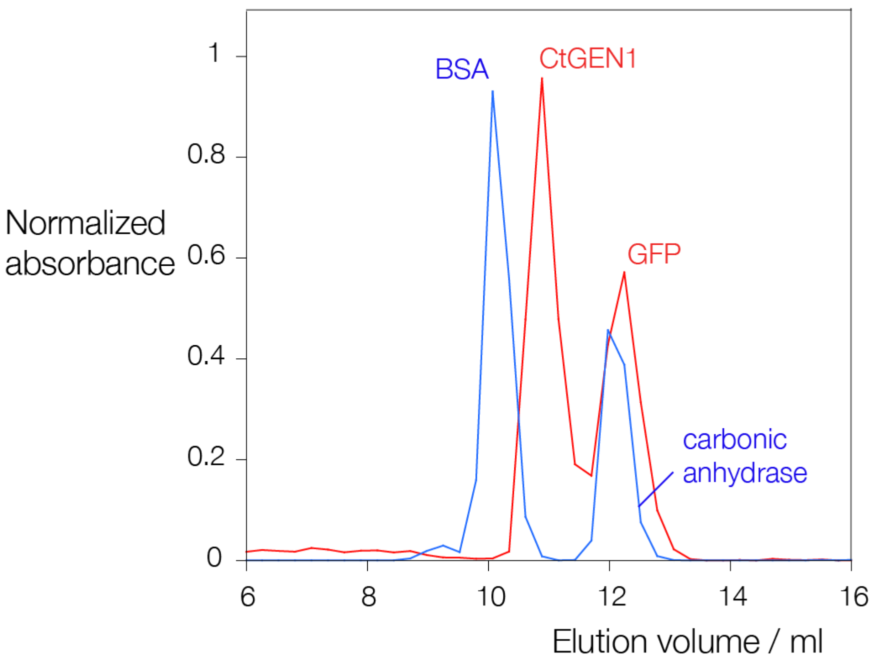

Supplementary Figure 5. Purified CtGEN1$_{1\text{-}487}$ was incubated with TEV protease to release it from the fusion with GFP and applied to a Superdex 75 10/300GL gel filtration column in 25 mM Hepes (pH 7.5), 50 mM NaCl (red line). Two peaks corresponding to CtGEN1$_{1\text{-}487}$ and GFP (28.4 kDa) are observed. BSA (66 kDa) and carbonic anhydrase (29 kDa) were applied to the same column as molecular mass standards (blue line). The calculated molecular mass of CtGEN1$_{1\text{-}487}$ is 55.1 kDa, so its migration as a single peak eluting between BSA and carbonic anhydrase indicates that it is monomeric in free solution. No elution was observed corresponding to a dimer of CtGEN1$_{1\text{-}487}$.

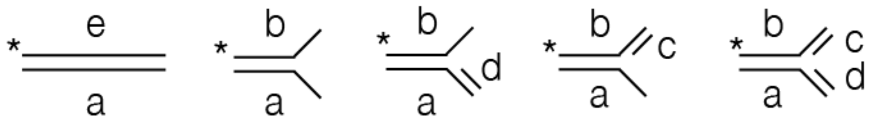

Construction of duplex, flap and nicked three-way junction substrates analysed in Figure 2.

a-strand (50 nt)
CCTCGATCCTACCAACCAGATGACGCGCTGCTACGTGCTACCGGAAGTCG

b-strand (50 nt)
CGACTTCCGGTAGCACGTAGCAGCGGCTCGCCACGAACTGCACTCTAGGC

c-strand (25 nt) GCCTAGAGTGCAGTTCGTGGCGAGC

d-strand (25 nt) CGTCATCTGGTTGGTAGGATCGAGG

e-strand (50 nt) CGACTTCCGGTAGCACGTAGCAGCGCGTCATCTGGTTGGTAGGATCGAGG

Junction J3 was assembled from four strands each of 50 nt :

b-strand GGCTAAGGGATCCGTCCTAGCAAGGGGCTGCTACCGGAGGCTTACATCGG

h-strand CCGATGTAAGCCTCCGGTAGCAGCCTGAGCGGTGGTTGGATGTTGACTGC

r-strand GCAGTCAACATCCAACCACCGCTCAACTCAACTGCAGTCTAGATGGACTG

x-strand CAGTCCATCTAGACTGCAGTTGAGTCCTTGCTAGGACGGATCCCTTAGCC

Junction Jbm5 was assembled from four strands each of 40 nt :

a-strand GCGTTACAATGGAAACTATTCGTGGCAGTTGCATCCAACG

b-strand CGTTGGATGCAACTGCCACGAATAGTGTCAGTTCCAGACG

c-strand CGTCTGGAACTGACACTATTCGTGGCGAATGGTCGTAAGC

d-strand GCTTACGACCATTCGCCACGAATAGTTTCCATTGTAACGC

Supplementary Figure 6. The nucleotide sequences of strands used to assemble the various substrates used in these studies. All sequences are written 5' to 3'.

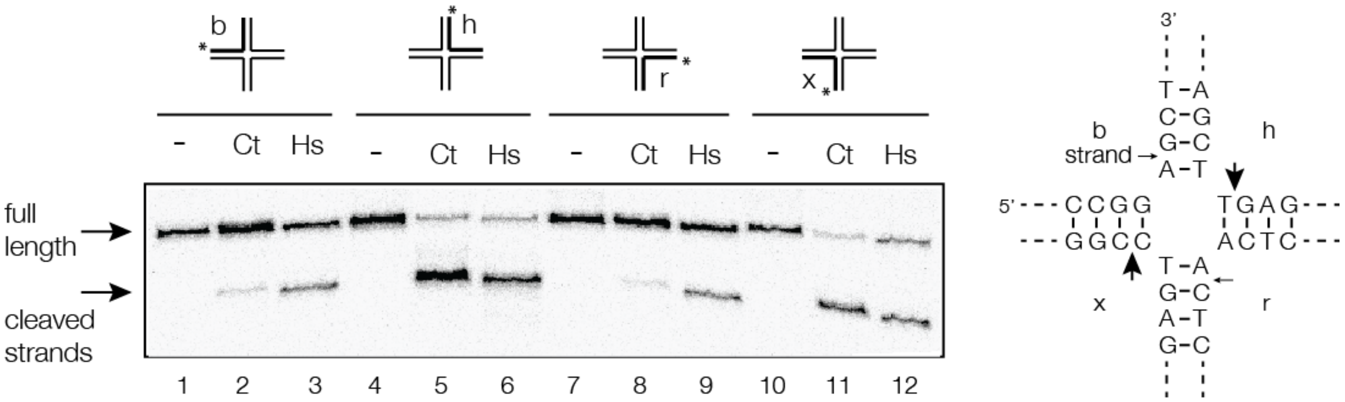

Supplementary Figure 7. Comparison of the pattern of cleavage of junction 3 by CtGEN-$1_{1\text{-}487}$ and human $GEN1_{1\text{-}527}$. The CtGEN1 cleavage products were generated as described in Figure 4. 5-10 nM of the same four junction 3 species radioactively [5'-^{32}P]-labeled on a single strand were incubated with 100 nM $HsGEN1_{1\text{-}527}$-GFP in 10 mM Tris (pH 8), 10 mM $MgCl_2$, 1mM DTT, 100 mM NDSB201, 10 μg/ml calf thymus DNA, 0.1 mg/ml BSA for 5 min. at 37ºC. Substrate and products were separated by electrophoresis in a 15% polyacrylamide gel containing TBE, 8 M urea at 80 W, and visualised by phosphorimaging. Tracks 1, 4, 7, 10 no enzyme added; tracks 2, 5, 8, 11 incubation with CtGEN-$1_{1\text{-}487}$; tracks 3, 6, 9, 12 incubation with $HsGEN1_{1\text{-}527}$-GFP. Junction 3 was radioactively [5'-^{32}P]-labeled on the b (tracks 1-3), h (tracks 4-6), r (tracks 7-9) or x strand (tracks 10-12).

$HsGEN1_{1\text{-}527}$-GFP was expressed and purified using the same procedure used to prepare CtGEN-$1_{1\text{-}487}$-GFP mutants. $HsGEN1_{1\text{-}527}$-GFP eluted from the heparin column at 0.6 M NaCl and was >95% pure, although some proteolytic products were present. Protein concentration was estimated by absorbance at 280 nm using A_{280} = 92,000 $M^{-1}cm^{-1}$.

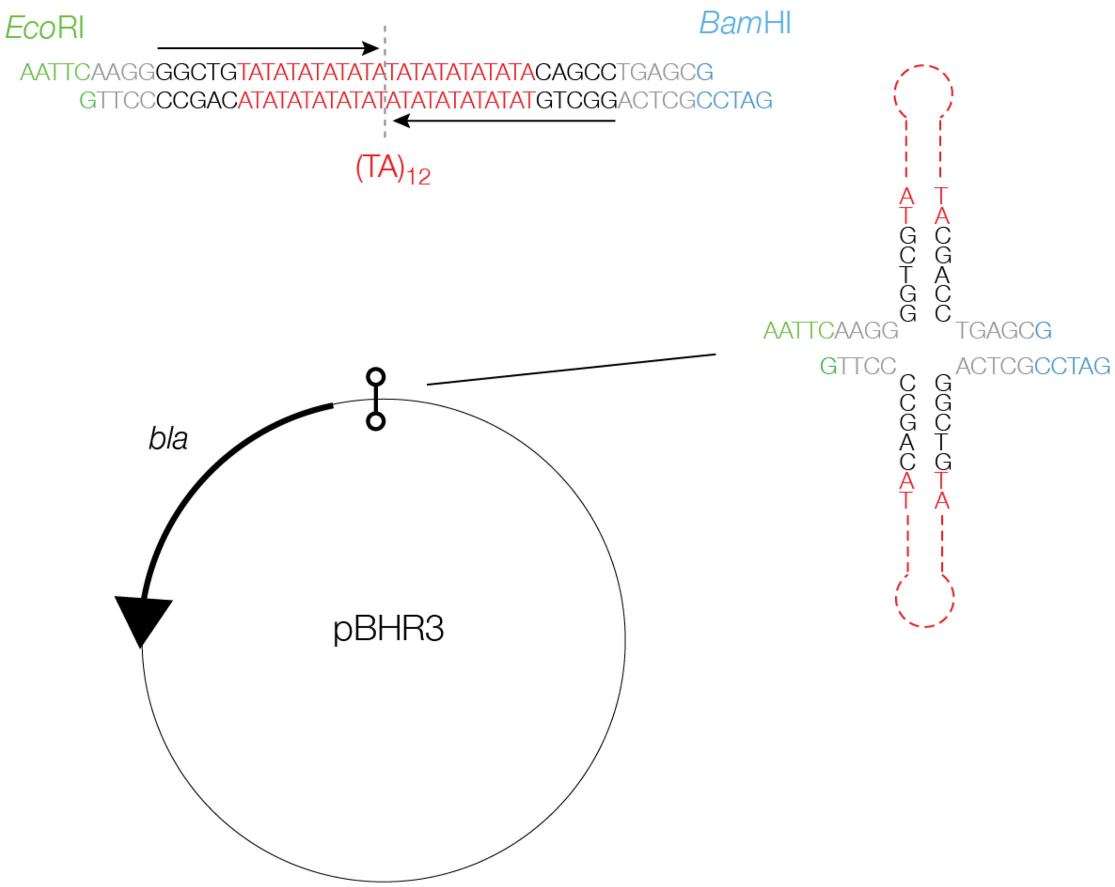

Supplementary Figure 8. Construction of the cruciform-containing plasmid pBHR3. The hybridized oligonucleotides shown (top left) were ligated between the *Eco*RI and *Bam*HI restriction sites of pAT153 to generate pBHR3, and transformed into *E. coli*. This extrudes the cruciform structure shown (right). This is formed from the 34 bp inverted repeat containing a central $(TA)_{12}$ sequence to ensure facile extrusion of the cruciform. The fully-extruded cruciform junction has a sequence corresponding to the B, H and R arms of junction 3.
